# A Strategy for Selective Deletion of Autoimmunity-Related T Cells by pMHC-Targeted Delivery

**DOI:** 10.3390/pharmaceutics13101669

**Published:** 2021-10-13

**Authors:** Shalom D. Goldberg, Nathan Felix, Michael McCauley, Ryan Eberwine, Lou Casta, Kathleen Haskell, Tricia Lin, Elizabeth Palovick, Donna Klein, Lori Getts, Robert Getts, Mimi Zhou, Pratima Bansal-Pakala, Vadim Dudkin

**Affiliations:** 1 Janssen Pharmaceuticals, Spring House, Montgomery, PA 19477, USA; nfelix4@ITS.JNJ.com (N.F.); MMcCaul3@its.jnj.com (M.M.); raeberwi@ITS.JNJ.com (R.E.); kmhaskell@verizon.net (K.H.); tricia.lin@live.com (T.L.); DKlein30@its.jnj.com (D.K.); pbansalp@its.jnj.com (P.B.-P.); dudkin@gmail.com (V.D.); 2 Genisphere LLC, Hatfield, PA 19440, USA; lou.casta@gmail.com (L.C.); epalovick@codebiotx.com (E.P.); lgetts@codebiotx.com (L.G.); bgetts@codebiotx.com (R.G.); 3 Janssen Pharmaceuticals, La Jolla, CA 92121, USA; MZhou@its.jnj.com

**Keywords:** peptide-MHC complexes, autoimmune disease, bioconjugates, 3DNA, nanomaterials

## Abstract

Autoimmune diseases such as rheumatoid arthritis are caused by immune system recognition of self-proteins and subsequent production of effector T cells that recognize and attack healthy tissue. Therapies for these diseases typically utilize broad immune suppression, which can be effective, but which also come with an elevated risk of susceptibility to infection and cancer. T cell recognition of antigens is driven by binding of T cell receptors to peptides displayed on major histocompatibility complex proteins (MHCs) on the cell surface of antigen-presenting cells. Technology for recombinant production of the extracellular domains of MHC proteins and loading with peptides to produce pMHCs has provided reagents for detection of T cell populations, and with the potential for therapeutic intervention. However, production of pMHCs in large quantities remains a challenge and a translational path needs to be established. Here, we demonstrate a fusion protein strategy enabling large-scale production of pMHCs. A peptide corresponding to amino acids 259–273 of collagen II was fused to the N-terminus of the MHC_II beta chain, and the alpha and beta chains were each fused to human IgG4 Fc domains and co-expressed. A tag was incorporated to enable site-specific conjugation. The cytotoxic drug payload, MMAF, was conjugated to the pMHC and potent, peptide-specific killing of T cells that recognize the collagen pMHC was demonstrated with tetramerized pMHC-MMAF conjugates. Finally, these pMHCs were incorporated into MMAF-loaded 3DNA nanomaterials in order to provide a biocompatible platform. Loading and pMHC density were optimized, and peptide-specific T cell killing was demonstrated. These experiments highlight the potential of a pMHC fusion protein-targeted, drug-loaded nanomaterial approach for selective delivery of therapeutics to disease-relevant T cells and new treatment options for autoimmune disease.

## 1. Introduction

Targeted therapeutics—modular conjugates or nanomaterials using targeting ligands for the delivery of therapeutic payloads to specific cellular addresses—is a field of great interest and growing prominence in the biopharmaceutical industry. The most prominent class of targeted therapeutics is antibody-drug conjugates (ADCs)—covalent conjugates of monoclonal antibodies (mAb) with cytotoxic small molecule drugs that are generally used for oncology applications [1]. The mAb component of ADCs binds to a cell-surface receptor that is overexpressed in cancer cells and that internalizes into endosomal compartments, allowing targeting of the drug payload to the interior of tumor cells. ADCs have been successful in the clinic, with 11 approved by the FDA and many more in clinical trials. Selective delivery of small molecule therapeutics to particular tissue or cell types is also a promising strategy in diseases beyond oncology, including cardiovascular disease [2], infectious disease [3], and immunological disorders [4].

Many immunological disorders arise from autoimmunity in which the immune system mounts an aberrant response to self-proteins. Peptides from self-proteins displayed on the surface of antigen-presenting cells by the major histocompatibility complex (MHC) are normally ignored by the immune system; however, in the case of autoimmunity inappropriate MHC recognition ultimately leads to the production of effector cells that recognize self-proteins, attack healthy tissue, and lead to disease progression. In many autoimmune disorders, one or a small number of MHC alleles are closely linked to incidence of disease, likely due to affinity of the critical peptide(s) for the MHC protein variant. In some diseases, post-translational modification on the peptides can also play a role in defining the binding affinity.

Rheumatoid arthritis (RA) is a systemic autoimmune disease that is characterized by chronic inflammation and progressive destruction of the synovial joints [5]. The autoimmune response in RA is further characterized by a strong MHC association (DRB1*04:01 is a risk allele for RA, for example) and T cell infiltration of inflamed joints [6]. Immune system recognition of joint proteins is believed to be a pivotal step in the development of RA and leads to degradation of joint tissue. CD4 T cell responses to a number of joint-relevant proteins have been identified in RA patients, but a definitive driver of disease remains unknown. One particularly well characterized autoantigen candidate is the 259–273 peptide from collagen II (CII_259). The CII_259 peptide binds to the DRB1*04:01 risk-allele [7] and T cell responses to this peptide have been reported in RA patients [8].

The treatments available for RA, while effective for some, still leave many patients inadequately controlled; moreover, all feature general immune suppression that has potential side effects including susceptibility to infection and cancer progression [9]. A treatment that could selectively clear T cell populations that recognize the critical self-antigens, such as CII_259, can be used to treat RA and/or pre-RA without the general immune system suppression that accompanies current treatments.

Peptide-loaded MHC complexes (pMHCs) drive the activation of T cells by binding to T cell receptors that are specific for these pMHCs. The affinity of an isolated T cell receptor for its cognate pMHC is typically relatively weak, generally >1 uM [10]. However, antigen-presenting cells (APCs) and T cells express many copies of the MHCs and TCRs respectively and the interaction is driven by avidity. Soluble pMHCs were first used to identify antigen specific T cell populations by binding biotinylated pMHCs to streptavidin-fluorophore conjugates to form tetramers [11], a technique that is now widely used. Building on pMHC tetramer staining technology, efforts have been made to develop pMHCs into targeting reagents that could selectively delete relevant T cell populations. By arming the pMHCs with cytotoxic payloads such as radionuclides [12] or bacterial toxins [13], it has been demonstrated that selective killing of T cells could be achieved in vitro and in a mouse model. Another approach that has been demonstrated is coating of the pMHCs onto nanoparticles which can potentially be tuned to induce outcomes ranging from T cell depletion to Treg expansion, among others [14,15].

In order to translate these approaches to a therapeutic platform, advancements are likely necessary. Peptide–MHC protein complexes can be difficult to produce in large quantities and often have limited shelf stability. Moreover, a strategy will be needed to efficiently conjugate a drug payload in a well-controlled manner without impacting the characteristics of the pMHC. An alternative multimerization approach is likely necessary due to immunogenicity risks associated with streptavidin [16], and it will need to be biocompatible, translatable, and tunable to allow adjustment of ligand density and payload loading.

Here, we describe the construction of a pMHC-II heterodimer fusion protein with handles for site-selective conjugation. The pMHCs were conjugated to cytotoxic drug and to biotin, resulting in tetramer complexes that demonstrated potent peptide-specific killing of T cell hybridoma lines. Finally, we describe production of 3DNA-based nanomaterials loaded with cytotoxic drug and targeted with pMHCs and demonstrate specific killing of T cell populations with these constructs.

## 2. Materials and Methods

### 2.1. Materials

Expifectamine transfection kit, polyacrylamide gels, biotin-NHS, Zeba columns, and AcTEV protease were from ThermoFisher. Growth media was from ThermoFisher or BD Difco. pAdvantage plasmid and CellTiterGlo were from Promega. MabSelect, HisTrap, Q Sepharose, and Superdex columns were from GE Healthcare. TSKGel column was from TOSOH. PNGase F was from NEB. Triglycine peptides were from Anaspec. Monomethyl auristatin F (MMAF) molecules were from Levena Biopharma. Modified oligonucleotides were from Oligo Factory or Biosynthesis.

### 2.2. Construction of Plasmids

Plasmids were constructed encoding proteins consisting of human HLA-DRA1:02 ECD (Genbank accession #EAX03629.1, amino acids 26–216) or peptide-DRB1*4:01 ECD (Genbank accession # QFI36205.1, amino acids 30–227) at the N-terminus, fused to the Fc domain of human IgG4 (Genbank accession #AAB59394.1 amino acids 105–326, modified with the “PAA” mutations [17]). In all cases, a TEV cleavage site (EDLYFQS) was encoded between the ECD and the Fc domain. The alpha chain plasmid included a C-terminal His_6_ tag and sortase tag (LPETGG). Genes also encoded the signal peptide from murine IgG for secretion and were placed under a CMV promoter.

### 2.3. Expression and Purification of Fusion Proteins

Proteins were expressed in HEK293 Expi cells using the ExpiFectamine transfection kit (Life technologies Cat# A14527 and A14524) following the manufacturing transfection protocol. Each of the plasmids encoding the Fc-fused alpha and beta chains, along with the pAdvantage plasmid to enhance protein expression (Promega Cat# E1711), were mixed with OptiMEM media. After 5 days total of incubation at 37 °C, 120 rpm, 8% CO_2_, supernatants were harvested by centrifugation for 30 min at 6000 rpm.

Proteins were purified on an AKTA Xpress chromatography system equipped with a 5 mL MabSelect Sure column. The column was equilibrated with 10 CVs of PBS buffer, pH 7.2, and supernatant was loaded onto column followed by washing with 10 column volumes of DPBS to remove unbound material. Protein was eluted with 0.1 M sodium acetate, pH 3.5 for 10 column volumes and the eluate collected in 1 mL fractions into 96-well blocks containing 0.2 volumes of 2 M Tris-HCl, pH 7. Peak fractions were pooled and dialyzed against 3 exchanges of Tris-buffered saline (50 mM Tris, 150 mM NaCl, pH 7.5). Concentration was measured by A280 using extinction coefficients calculated from the protein sequences.

### 2.4. Biotin Conjugation

pMHC fusion proteins were biotinylated by two different methods. For random conjugation to lysine residues, pMHC proteins at 1 mg/mL (9.5 μM) were incubated with a 12-fold excess of biotin-NHS in PBS buffer supplemented with 100 mM sodium bicarbonate pH 9 for 20 min at RT. After quenching with 1 M Tris-HCl pH 7.5, the protein was purified by size-exclusion chromatography on an AKTA Avant equipped with a Superdex 200 Increase 10/300 GL column run at 0.5 mL/min using PBS mobile phase. Fractions containing protein were pooled and concentrated with an Amicon centrifuge concentrator with 50 kDa MWCO.

For sortase-catalyzed transpeptidation, the *S. aureus* enzyme with 5 mutations that increase catalytic efficiency [18] and lacking the N-terminal membrane anchoring region was used. pMHC fusion protein (9.7 μM) was combined with a ~10-fold molar excess (95 uM) of the tetrapeptide Gly-Gly-Gly-biotinyl lysine and 0.2 μM sortase in a reaction containing 50 mM Tris-HCl pH 7.5, 150 mM NaCl, 10 mM CaCl_2_. After 2.5 h incubation at RT with gentle rocking, the reaction was stopped by the addition of EDTA to 100 mM. The conjugated pMHC was purified by size-exclusion chromatography as above.

### 2.5. MMAF Conjugation

For random conjugation of the MMAF drug payload, the pMHC proteins were first reacted with NHS-azide to attach azide functional groups to lysine sidechains. Proteins at 0.85 mg/mL (8 μM) were incubated with a 6-fold excess of NHS-azide in PBS buffer supplemented with 100 mM sodium bicarbonate buffer, pH 9 for 10 min at RT. After quenching with 1 M Tris-HCl pH 7.5, the protein was exchanged into PBS. A 20-fold excess of DBCO-PEG4-vc-PAB-MMAF was added and mixture was incubated for 1 h at 37 °C. The conjugate was exchanged into PBS, concentrated with Amicon concentrators, and characterized by MS and SEC.

### 2.6. Protein Characterization Methods

Purity of proteins and conjugates was assessed by running 2 μg of reduced and non-reduced protein on 4–12% Bis-Tris SDS-PAGE gels. For LC-MS, the sample was denatured with 4M guanidinium hydrochloride followed by desalting with Zeba columns and overnight treatment with PNGase F at 37 °C followed by intact mass analysis on a Sciex X500B instrument or an Agilent Model G6224 MS-TOF instrument equipped with an Agilent RP-mAb C4 2.1 × 50 mm, 3.5 micron column. Alternatively, the sample was cleaved with AcTEV protease and then treated with PNGase F prior to LC-MS. For the TEV method, the alpha/beta heterodimer was generally not detected; thus, only the mass of the Fc fragment was assessed; where relevant, DOL was extrapolated to the full molecule.

Oligomerization state was determined by HPLC-SEC using a TSKgel BioAssist G3SWxl column (7.8 mm × 30 cm, 5 μm) with DPBS mobile phase run at 1 mL/min.

### 2.7. 3DNA Synthesis

Essentially, 3DNA nanomaterials were produced as described [19,20]. Briefly, seven unique DNA strands were pairwise hybridized to form branched monomer structures comprising a double stranded portion and 4 single strand arms, two 5’ and two 3’. The hybridization complementarity of these arms was used to prepare a layered combination of DNA monomers resulting in a DNA matrix having a core of double stranded DNA and 36 single stranded arms on the surface split equally between 5’ and 3’ ends. These 5’ and 3’ arms (18 each) were hybridized to oligonucleotides coupled to targeting moieties and drugs.

The 5’-arm drug-oligo conjugate containing a single MMAF was prepared using thiol/maleimide chemistry. Eight mg of 5’-arm thiol oligo was reduced using 50 mM TCEP and purified via ethanol precipitation. The reduced oligo (6.4 mg) was conjugated to maleimido-PEG_4_-vcMMAF by incubating with a 10-fold molar excess of drug in 1× PBS overnight at RT. MMAF-oligo conjugate was purified via ethanol precipitation and analyzed via denaturing gel electrophoresis and RP-HPLC. Peak areas were used to calculate the ratio and determined to be 0.84 drugs per oligo. 

The 5’-arm drug oligo conjugate containing 2 molecules of MMAF per oligo was prepared using DBCO click chemistry. The 5’-arm dual amine oligo was modified with ~200 fold molar excess azido-PEG_4_-NHS (Click Chemistry Tools), incubated at RT for 1 h and then placed at 4 °C overnight. Excess azido-PEG_4_-NHS was removed by ethanol precipitation and analyzed via denaturing gel electrophoresis. The 5’ dual azide oligo (600 μg) was then conjugated to DBCO-PEG4-vc-PAB-MMAF (~50 fold molar excess) by incubation at room temperature for 1 h followed by 4 °C overnight. The dual MMAF-oligo conjugate was purified via ethanol precipitation and analyzed via gel electrophoresis and RP-HPLC. Peak areas were used to calculate the ratio and determined to be 1.7 drugs per oligo.

Drug–oligo conjugates were analyzed by reversed-phase HPLC (Agilent 1100) using a Zorbax 300SB-C18 column (Agilent) with mobile phases 0.1 M triethylammonium acetate pH 6.6 (HA) and acetonitrile (HB). The following elution profile was utilized for analysis: 0–5 min 90% HA, 10% HB with HB increasing from 10–20% at a flow rate of 1 mL/min; then 5–20 min HB increasing from 20–70% at a flow rate of 1 mL/min; then 20–27.3 min with 100% HB at 1 mL/min; then 27.3–34.3 min with 90% HA 10% HB at 1 mL/min. Elution of the drug, oligo and drug-oligo conjugates were monitored by UV absorbance at 260, 280 and 300 nm.

DBCO 3’-arm conjugate was prepared by reacting an amine on the 3DNA 3’-arm oligo (20 mg, Oligo Factory) with ~50 fold molar excess NHS-PEG_5_-DBCO (Click Chemistry Tools), incubated overnight at room temperature and purified via ethanol precipitation to remove excess unreacted NHS-PEG_5_-DBCO, and analyzed via denaturing gel electrophoresis.

The pMHCs were conjugated to azide using sortase-catalyzed transpeptidation. pMHC fusion protein (10 μM) was combined with a ~10-fold molar excess (95 μM) of the peptide H-Gly-Gly-Gly-PEG_4_-azide and 0.2 μM sortase in a reaction containing 50 mM Tris-HCl pH 7.5, 150 mM NaCl, 10 mM CaCl_2_. After 2.5 h incubation at RT, the reaction was purified by nickel affinity chromatography on an AKTA Purifier equipped with a HisTrap HP column. Reaction was loaded onto the column, washed with 5 CVs of wash buffer (50 mM Tris pH 7.5, 0.5 M NaCl, 10 mM imidazole) and eluted with 5 CVs of elution buffer (50 mM Tris pH 7.5, 250 mM imidazole).

Azide-modified pMHCs were conjugated to the 3’ DBCO-modified oligo at 37 °C. Oligo at 36.4 μM and pMHC at 14.6 μM were combined in a reaction mixture containing 25 mM Tris pH 7.5, 125 mM imidazole. Following overnight incubation, pMHC-oligo conjugate was purified in two steps. Unconjugated protein was removed by ion exchange chromatography on an AKTA Avant equipped with a 1 mL Q Sepharose column. Column was equilibrated and washed in TBS buffer (50 mM Tris pH 7.5 150 mM NaCl) followed by elution with a 0–100% gradient of 50 mM Tris pH 7.5, 1.2 M NaCl over 50 CVs. Fractions containing conjugate were purified further to remove free oligo by SEC on a HiLoad 16/600 Superdex 200 pg with PBS mobile phase run at 0.5 mL/min.

The 3DNA:MMAF:pMHCs were assembled by first preparing master mixes of the 3DNA drug oligo conjugate. 3DNA and drug oligo conjugate (one or 2 drugs per oligo) were combined in 1X PBS pH 7.4 and incubated for 30 min at 37 °C. These master mixes (3DNA-MMAF15, 3DNA-MMAF31) were then combined with the oligonucleotide conjugated pMHC proteins and incubated at 37 °C for 30 min, to produce the 6 formulations for each pMHC at the final components concentrations as listed in Table 1.

The hydrodynamic diameter (Z avg) and polydispersity index (PDI) of the 12 different formulations were determined by dynamic light scattering on a Zetasizer Nano ZS (Malvern Instruments, Malvern, UK). Each sample (40 uL at 250 ng/uL (as pMHC protein)), was analyzed in quadruplicate and the mean values +/− standard deviation are reported in Table 1.

### 2.8. Generation of T cell Hybridomas

The CII.DR4.23.5 hybridoma was a generous gift from Ed Rosloniec. The HA.5D3.9 and CII.26B6.18 hybridomas were generated using standard techniques [21]. In brief, HLA-DR4 transgenic mice (Taconic Biosciences, Germantown, NY, USA) were immunized in the hock with HA or Col_II peptide (Genscript, Piscataway, NJ, USA) in complete Freund’s adjuvant (BD Difco). Draining lymph node cells (popliteal and inguinal) and splenocytes were recovered 1 week later and cultured in medium with 10 uM peptide for 4 days. Cells were then purified using Lympholyte-M (Cedarlane, Burlington, ON, Canada), washed with HBSS and cultured in medium with 100 U/mL IL-2 for an additional 3 days. The cells were then fused at a 1:1 ratio with the BW5147α-β- fusion partner (National Jewish Health). After fusion, cells were cultured in Iscove’s medium containing 20% FCS and hypoxanthineaminopterin-thymidine. Growth positive wells were tested for reactivity and specificity by culturing with DR4+ BOLETH APCs (Fred Hutchinson Cancer Research Center IHWG Cell and Gene Bank) with or without 10 uM cognate peptide (HA or Col_II, as appropriate) as assaying for IL-2 production (AlphaLISA; Perkin Elmer, Waltham, MA, USA). Reactive cells were subcloned to ensure monoclonality.

### 2.9. Tetramer Formation and FACS

Peptide–MHC tetramer binding to cognate T-hybridoma was assessed by flow cytometry. Tetramers were prepared via five equal additions of streptavidin-phycoerythrin (Prozyme, Hayward, CA USA) totaling an equimolar amount to biotinylated pMHCs. For tetramer binding assays, 150,000 cells were plated per 96-well plate and pre-treated with 50 nM dasatinib (Bio Vision Inc., Milpitas, CA, USA) for 30 min at 37 °C in complete media. Media formulation for HA5D3.9 and Col_II-26.B6.18 is IMDM supplemented with 20% FBS and for DR4.CII.23.5 is DMEM supplemented with 10% FBS, Glutamax, pen-strep, and 50 mM beta-mercaptoethanol. Tetramers were then added at indicated concentrations and incubated for 1 h at room temperature. Anti-CD3-APC (Biolegend, San Diego, CA USA) was then added and incubated for 30 min at 4 °C. Cells were washed 1× in PBS/BSA before fixing for 10 min at room temperature with Cytofix (BD). Data were acquired on the CytoFLEX LX (Beckman-Coulter, Brea, CA USA) and analyzed with Cytobank software.

### 2.10. Cytotoxicity Assays

Cell killing was assessed by measuring viability of the indicated T cell hybridoma following exposure to the conjugates. Cells were plated in black-well, clear bottomed, tissue culture-treated plates at 1500 cells/well in 60 μL/well of the appropriate growth medium. Cells were allowed to settle overnight at 37 °C in a humidified 5% CO_2_ atmosphere. and then treated with 60 μL of 2× concentrations of test article in fresh media. Cell viability was determined by an endpoint assay with Cell TiterGlo after 3 days. Data were calculated as mean ± standard deviation of at least 2 replicates. IC_50_ values were determined by fitting data to the equation for a sigmoidal dose response using GraphPad Prism software.

## 3. Results and Discussion

### 3.1. Design of pMHC Fc Fusion Proteins

Monomers for peptide–MHC class II tetramers have been produced in a variety of systems including insect cells and E. coli. Bacterial expression is most commonly used and involves expressing the alpha and beta MHC subunits in inclusion bodies, purification in a denatured state, and then refolding the proteins in the presence of chemically synthesized peptide [22]. Finally, another purification step is needed before biotinylation. The process is involved and low yielding, and the resulting pMHCs have limited stability. Insect cell expression has also met with complications and has similar challenges with process complexity and yield [23]. To enable rapid and efficient production of more stable pMHC complexes, a fusion protein strategy was implemented.

**Figure 1:**
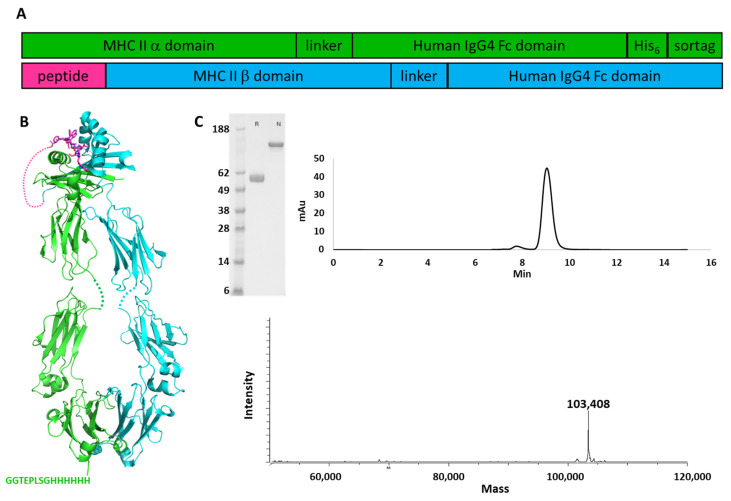
Production of pMHC fusion proteins. (**A**) Arrangement of alpha and beta domain fusion genes (**B**) Cartoon schematic of assembled pMHC-Fc fusion. Alpha chain Fc fusion is shown in green and beta chain Fc fusion in cyan. pMHC structure from 5V4N [25]; Fc structure from 3D6G [26]. (**C**) Characterization of pMHC-Fc fusion protein. SDS-PAGE, reduced (R) and non-reduced (N); analytical SEC; and mass spectrometry of the Col_II pMHC protein are shown.

The extracellular domain of HLA-DRA1 was fused to the Fc region of human IgG4, and a His_6_ tag was placed at the C-terminus to enable purification, followed by the sortase recognition sequence for conjugation [24]. The DRB1 ECD (DRB1*04:01) was fused to the CII_259 peptide corresponding to amino acids 259–273 of human collagen II, or to a control peptide from influenza hemagglutinin (amino acids 306–318), at its N-terminus. The CII peptide has relatively weak affinity for the MHC complex, on the order of 1 uM [7], and will dissociate from a purified complex over time. Thus, the peptide was covalently tethered to the MHC subunit to maintain stability with a 14-amino acid linker (Figure 1).

The α and β chains were co-expressed in HEK293 Expi cells transfected transiently with the encoding plasmids. After 5 days of expression, protein was collected from the culture media and purified by protein A affinity chromatography. The heterodimer was the only product recovered and no further purification was needed (Figure 1C). Purity of the proteins was demonstrated by SDS-PAGE, in which only the band corresponding to intact fusion protein is visible in the non-reduced sample; analytical SEC showing a major peak (area > 95%) corresponding to the heterodimer; and mass spectrometry which yielded the mass corresponding the intact heterodimer. Similar results were obtained with the pMHC_HA fusion (data not shown). This suggests that association of the alpha and beta MHC subunits alone is sufficient to favor heterodimer formation over homodimer formation of either of the chains and no mutations need be incorporated into the Fc domain to drive heterodimer pairing. Furthermore, a second chromatography step appears to be unnecessary to obtain high purity for these molecules, suggesting that any homodimers and monomers that may be produced are sufficiently unstable that they do not remain in the soluble fraction. Final yields of 30–55 mg of protein per liter of culture were obtained. Oligomerization state was determined by analytical size exclusion and the proteins were determined to be >95% monomeric. 

### 3.2. Optimization of Conjugation Scheme

In order to produce conjugates featuring both biotin for tetramerization and the therapeutic drug payload, sequential conjugation steps were employed. Biotinylation was performed first, and two approaches were tested: random conjugation to lysines using a commercial NHS-biotin reagent or site-specific enzymatic conjugation with sortase. The random approach resulted in conjugates with an average degree of labeling (DOL) estimated to be ~4 for the entire alpha/beta heterodimer. The sortase reaction produced conjugates with DOL exactly 1, at the C-terminus of the alpha chain. In both cases, a higher-MW species was observed by analytical SEC, ranging from 8–15% (data not shown).

**Figure 2:**
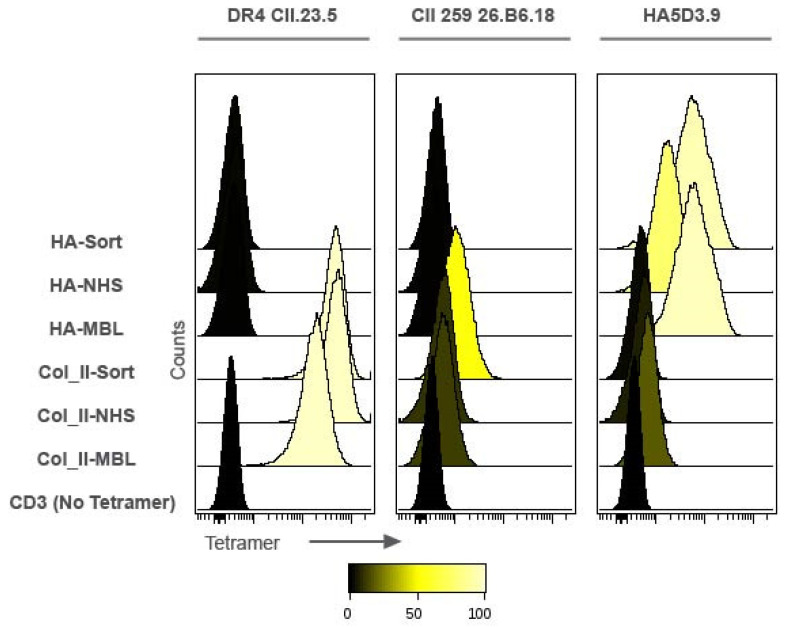
Impact of biotinylation strategy on pMHC tetramer binding. Cells were stained with tetramerized pMHCs at 187.5 nM monomer concentration and analyzed by flow cytometry. Shown are the apparent high affinity collagen II hybridoma line CII.DR4.23.5; the apparent lower affinity CII.26B6.18 hybridoma line; and the HA peptide specific line HA5D3.9. Site-specific conjugates (HA-Sort and Col_II-sort) are compared to random conjugates (HA-NHS and Col_II-NHS); commercial pMHC tetramers (MBL) were included as a positive control, and cells treated with CD3 only and no tetramer as a negative control.

The biotinylated pMHCs were tetramerized with streptavidin-phycoerythrin in equimolar ratios and binding of the tetramers to cell lines specific for the Col_II peptide (DR4.CII.23.5 and CII.26.B6.18) or the HA peptide (HA.5D3.9) was assessed by flow cytometry. Both Col_II tetramers showed binding to the DR4.CII.23.5 cell line; meanwhile in the CII.26B6.18 cells that generally showed weaker staining, the Col_II tetramer that was biotinylated by sortase had the higher signal in terms of MFI and % staining. The Col_II tetramers had essentially background levels of binding to the influenza HA-specific cell line, HA.5D3.9. The HA tetramer biotinylated with sortase had the strongest signal of these molecules in the HA-specific cell line HA.5D3.9, and both HA tetramers had negligible binding to Col_II specific cell lines (Figure 2).

Monomethyl auristatin F (MMAF) [27] was selected as the drug payload due to its validation as a payload for ADCs, sub-nM potency, and limited cell permeability—thus requiring receptor-mediated internalization to achieve cell killing and minimizing bystander killing and off-target toxicity. The well-established protease cleavable linker valine-citrulline-para-aminobenzyl carbamate (VC-PAB or vc) [28] was chosen to enable drug release in endosomal compartments. 

To produce the drug conjugates, the pMHCs were biotinylated via the sortase tag and then conjugated to drug. Azide groups were added to lysine sidechains by reacting the proteins with NHS-azide. This was followed by strain-promoted azide-alkyne cyclization (SPAAC) reaction to attach DBCO-vcMMAF to the azido groups on the protein. The pMHCs were modified to approximate drug-antibody ratio (DAR) of 2.7 as determined by LC-MS (Figure S1).

**Figure 3:**
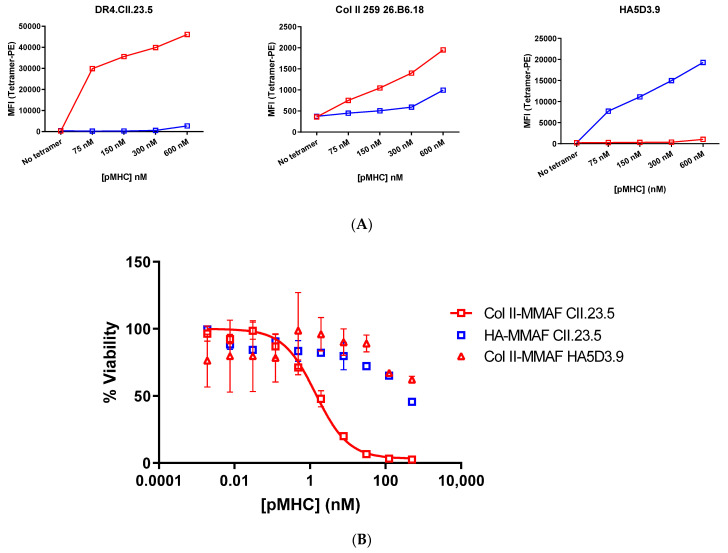
Activity of drug-loaded pMHC tetramers. (**A**) Binding of random and site-specific conjugates to hybridoma cell lines DR4.CII.23.5 specific for pMHC_Col_II-MMAF tetramers (red) and HA5D3.9 specific for pMHC_HA-MMAF tetramers (blue) was determined by FACS. Cells were stained with varying concentrations of streptavidin-PE tetramerized pMHC drug conjugates and analyzed by flow cytometry (**B**) Cell lines were treated with varying concentrations of tetramerized pMHC-MMAF conjugates constructed with the CII peptide fusion or the HA peptide fusion. Cells were treated for 72 h and then viability was assessed with Cell Titer Glo.

The biotinylated and drug conjugated pMHCs were then tetramerized by the addition of streptavidin-PE and tetramer binding to cognate cell lines was assessed by FACS. The HA pMHC bound to the HA cell line at all concentrations tested and showed weak non-specific binding to the Col_II cell lines at the highest concentrations. The Col_II pMHC showed a strong binding signal with the DR4.CII.23.5 cell line, and a weaker signal on the CII.26B6.18 line (Figure 3a). The tetramers were then evaluated for specific killing of Col_II positive cell lines.

### 3.3. pMHC-Tetramer Cell Killing Assays

To evaluate targeted delivery of therapeutic payload to peptide-specific T cells using pMHC-tetramers, the hybridoma cell line that specifically recognizes the Col_II peptide was treated with the drug-loaded tetramers. DR4.CII.23.5 cells were treated with the random and site-specific tetramerized drug conjugates at concentrations ranging from 2 pM to 500 nM of the pMHC-Fc protein for 72 h. The tetramers formed from the CII pMHC drug conjugates showed potent cell killing, with IC50 of 1.4 nM and maximum cell killing >99%, while the HA pMHC conjugates had limited potency, only barely exceeding 50% cell killing at the highest 500 nM concentration (Figure 3b). Thus, potent, peptide-specific cell killing was observed in the cell line that recognizes Col_II peptide-loaded MHCs, while little toxicity was observed with drug conjugates lacking the Col_II peptide. The Col_II pMHCs also showed limited cell killing in the HA-specific T cell line. Taken together, we see potent activity suggesting a wide therapeutic window between killing of the target cells and of non-target T cell populations.

### 3.4. Production of 3DNA-pMHC Drug Loaded Assemblies

In order to establish a more translatable therapeutic platform, the biotin-streptavidin component of the pMHC-targeted drug conjugates was replaced with a nanomaterial strategy. Since we were focused on using a highly defined platform that would allow tight control of the targeting ligand density and drug load, 3DNA^®^ technology was selected.

**Figure 4:**
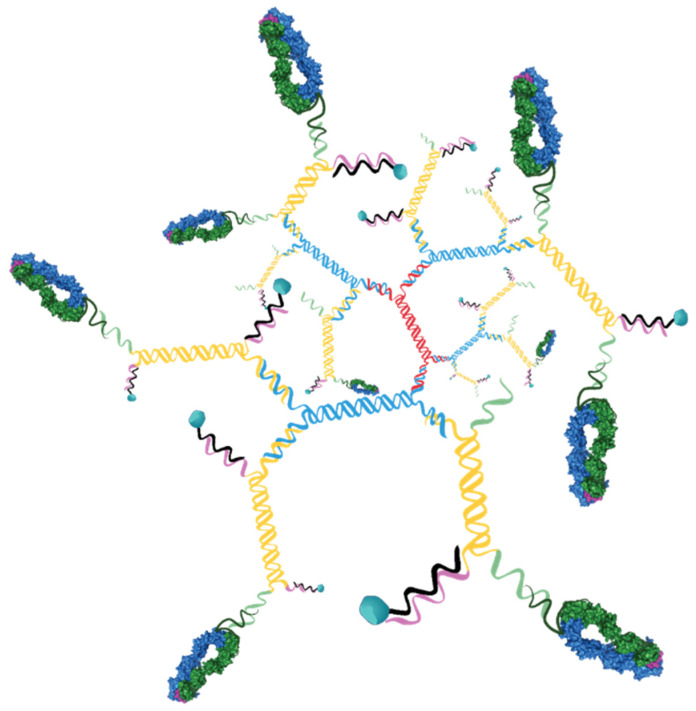
Schematic of 3DNA nanomaterials. Cartoon representation of 3DNA with 9 targeting ligands and 18 drugs is shown.

This 3DNA is a nanocarrier platform that is composed of DNA strands, hybridized and crosslinked to form branched assemblies. Attachment of targeting ligands and payloads to the DNA strands allows for assembly of precisely defined particles with well controlled ligand and payload densities (Figure 4).

To produce 3DNAs with pMHC targeting ligands and vcMMAF drug payload, first 3DNA was manufactured as described [19,20] to yield a DNA matrix having a core of double stranded DNA and 36 single stranded arms on the surface split equally between 5’ and 3’ ends. The 5’ arms were hybridized to oligonucleotides that contained the drug payload and the 3’ arms were hybridized to oligos that were conjugated to targeting ligand. 

The 5’-arm drug–oligo conjugates were prepared containing one or two molecules of MMAF per oligo using thiol/maleimide chemistry and SPAAC chemistry respectively. These oligos were hybridized to the 18 5’ ends of the 3DNA to yield particles with a theoretical maximum of 18 or 36 MMAF molecules per particle. Analytical characterization of the batches determined that the actual drug loads were 15 and 31 MMAF molecules per particle.

The 3’-arm oligo was modified with DBCO and then conjugated to azide-modified pMHC proteins that were generated by sortase-catalyzed transpeptidation of the pMHCs with the azide-containing triglycine peptide Gly-Gly-Gly-PEG_4_-azide (Figure S2). These were hybridized to the 3DNA at a ratio designed to obtain the desired ligand density.

For each pMHC, six formulations of 3DNA:MMAF:pMHC were produced with different combinations of ligand density (18, average of 9, or average of 4 per particle) and drug load (15 or 31 per particle—Table 1). The 3DNA assemblies were characterized by dynamic light scattering and the mean diameter (Z-avg) was found to range from 65 to 83 nm, with polydispersities between 0.13 and 0.22. Overall, the 15-drug assemblies had larger diameters measured than the 31-drug assemblies (79.0 nm and 71.4 nm averages, respectively) and the 18-pMHC assemblies (80.3 nm average) had larger diameter than the 9-pMHC (73.4) or 4-pMHC (71.8) assemblies.

### 3.5. 3DNA Cell Killing Assays

**Figure 5:**
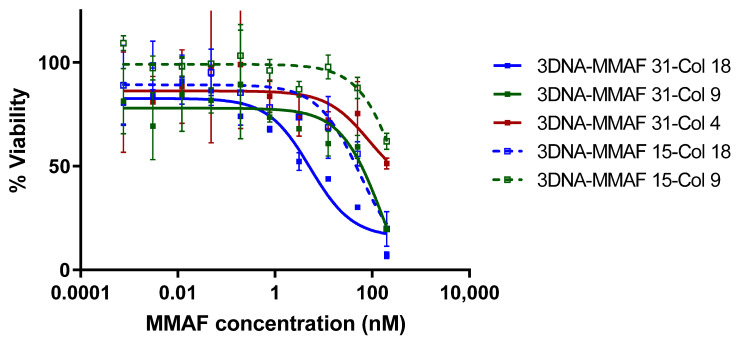
Cytotoxicity of drug-loaded pMHC-3DNAs. DR4.CII.23.5 hybridoma cell line specific for Col_II_259 peptide was treated with varying concentrations of vcMMAF-conjugated pMHC-3DNA nanomaterials constructed with the CII peptide fusion. Cells were treated for 72 h and then viability was assessed with Cell Titer Glo. 3DNA-MMAF 15-Col 4 particles showed no significant killing at any concentration and data is omitted for clarity.

To evaluate peptide-specific T cell targeting with each of the 3DNA formulations, the Col_II peptide specific hybridoma cell line DR4.CII.23.5 was treated with drug-loaded 3DNAs. Cells were treated with the 3DNA assemblies at MMAF concentrations ranging from 1 pM to 200 nM for 72 h. The 3DNAs targeted with HA pMHC ligands showed no activity at the concentrations tested regardless of ligand density or drug load. The Col_II pMHC-targeted 3DNAs had a range of activity that was dependent on both ligand density and drug load (Figure 5). For the particles with drug load of 31, the highest density of pMHC gave an IC50 of 3.3 ± 1.8 nM over two replicates, with maximal cell killing of 93% at the highest concentration tested. This potency is comparable to that observed with the tetramer drug conjugates, which had an IC50 of 3.8 nM based on MMAF (taking into account the degree of labeling of 2.7). Meanwhile, the medium and lowest pMHC density were significantly less potent (53.4 ± 7.3 and >100 nM respectively). The particles with drug load of 15 were less potent overall, and also showed ligand density dependence. The highest pMHC density particles had an IC50 of 29.9 ± 17.1 nM, medium density showed some activity (~40% cell killing) only at the highest concentration tested, and the lowest density particles had no detectable activity at the concentrations tested.

The targeted delivery of drug-conjugate to cell lines with TCRs that recognize the collagen peptide-MHC complex demonstrate the potential for using this approach to selectively delete or otherwise modify pathogenic T cell populations without affecting normal immune system function. Additionally, given the modularity and flexibility of the 3DNA platform, there are many avenues to optimize the 3DNA-based delivery system. One approach would be to increase the drug loading, since the data suggest higher drug load per assembly (31 drugs/molecule) correlates to an increased potency. The 3DNA particles could be loaded with greater than 100 drugs per particle by coupling DBCO-PEG4-VC-PAB-MMAF to a 3DNA binding oligonucleotide containing six or more azides per oligo. Another path to improving delivery could be via altering the pMHC ligand arrangement on the particle. While additional ligand density is likely impractical, changing the attachment of pMHC ligands to the 3DNA could provide a boost to cell binding by increasing the avidity. For example, the ligands can be localized to one region of the 3DNA thereby increasing the local density. Orientation of the pMHCs on the assembly can be tailored in a number of other ways, such as attaching two or more at each 3DNA arm in an effort to increase the avidity effect.

Additionally, these experiments were conducted with MMAF, a tubulin inhibitor designed for cancer treatment. Exploring different therapeutic payloads that may be more specific to T cells is warranted and may be a better approach. Nucleic acid therapeutics such as siRNA and antisense may also prove to be a useful way of treating the T cell populations with reduced risk of cytotoxicity in healthy tissue.

In conclusion, we have demonstrated an efficient method of producing stable peptide-MHC fusion proteins. Upon multimerization, these pMHCs bind specifically to cell lines with the corresponding T cell receptors, internalize into the cells, and deliver a payload. This technology points the way toward a modular, translatable platform (Targeted 3DNA) for selective deletion of T cells that are drivers of autoimmunity, an approach that can provide for safer therapeutics to this class of diseases.

## Figures and Tables

**Table 1:** Characteristics of 3DNA assemblies. Samples were formulated at 1 μM MMAF in PBS. Mean particle diameter was determined by dynamic light scattering.

Sample	Drugs per 3DNA	pMHCs per 3DNA	Z-Avg (nm)	PDI
3DNA-MMAF 15—pMHC CII 18	15	18	81.17 +/− 0.67	0.145 +/− 0.008
3DNA-MMAF 15—pMHC CII 9	15	9	80.56 +/− 2.71	0.201 +/− 0.020
3DNA-MMAF 15—pMHC CII 4	15	4	75.98 +/− 1.58	0.201 +/− 0.016
3DNA-MMAF 15—pMHC HA 18	15	18	81.68 +/− 0.94	0.155 +/− 0.007
3DNA-MMAF 15—pMHC HA 9	15	9	75.67 +/− 1.09	0.169 +/− 0.014
3DNA-MMAF 15—pMHC HA 4	15	4	78.81 +/− 1.97	0.218 +/− 0.014
3DNA-MMAF 31—pMHC CII 18	31	18	75.35 +/− 0.36	0.134 +/− 0.012
3DNA-MMAF 31—pMHC CII 9	31	9	67.92 +/− 0.75	0.132 +/− 0.014
3DNA-MMAF 31—pMHC CII 4	31	4	67.25 +/− 3.51	0.18 +/− 0.029
3DNA-MMAF 31—pMHC HA 18	31	18	83.15 +/− 1.19	0.212 +/− 0.008
3DNA-MMAF 31—pMHC HA 9	31	9	69.43 +/− 1.38	0.152 +/− 0.024
3DNA-MMAF 31—pMHC HA 4	31	4	65.29 +/− 1.55	0.184 +/− 0.020

## Data Availability

The data presented in this study are available on request from the corresponding author.

## Supplementary Materials

The following are available online at https://www.mdpi.com/article/10.3390/pharmaceutics13101669/s1, Figure S1: Random conjugation of NHS-azide. Figure S2: Characterization of pMHC-oligonucleotide conjugates.
