# Supplementary material for: A Strategy for Selective Deletion of Autoimmunity-Related T Cells by pMHC-Targeted Delivery
Source: Pharmaceutics. 2021 Oct 13;13(10):1669. doi: 10.3390/pharmaceutics13101669 (PMC8540115; doi:10.3390/pharmaceutics13101669)
Supplement: Supplementary file 1 [file pharmaceutics-13-01669-s001.zip › pharmaceutics-1384210-supplementary.pdf]

pharmaceutics
MDPI

# Supplementary Materials: A Strategy for Selective Deletion of Autoimmunity-Related T cells by pMHC-Targeted Delivery

**Shalom D. Goldberg, Nathan Felix, Michael McCauley, Ryan Eberwine, Lou Casta, Kathleen Haskell, Tricia Lin, Elizabeth Palovick, Donna Klein, Lori Getts, Robert Getts, Mimi Zhou, Pratima Bansal-Pakala and Vadim Dudkin**

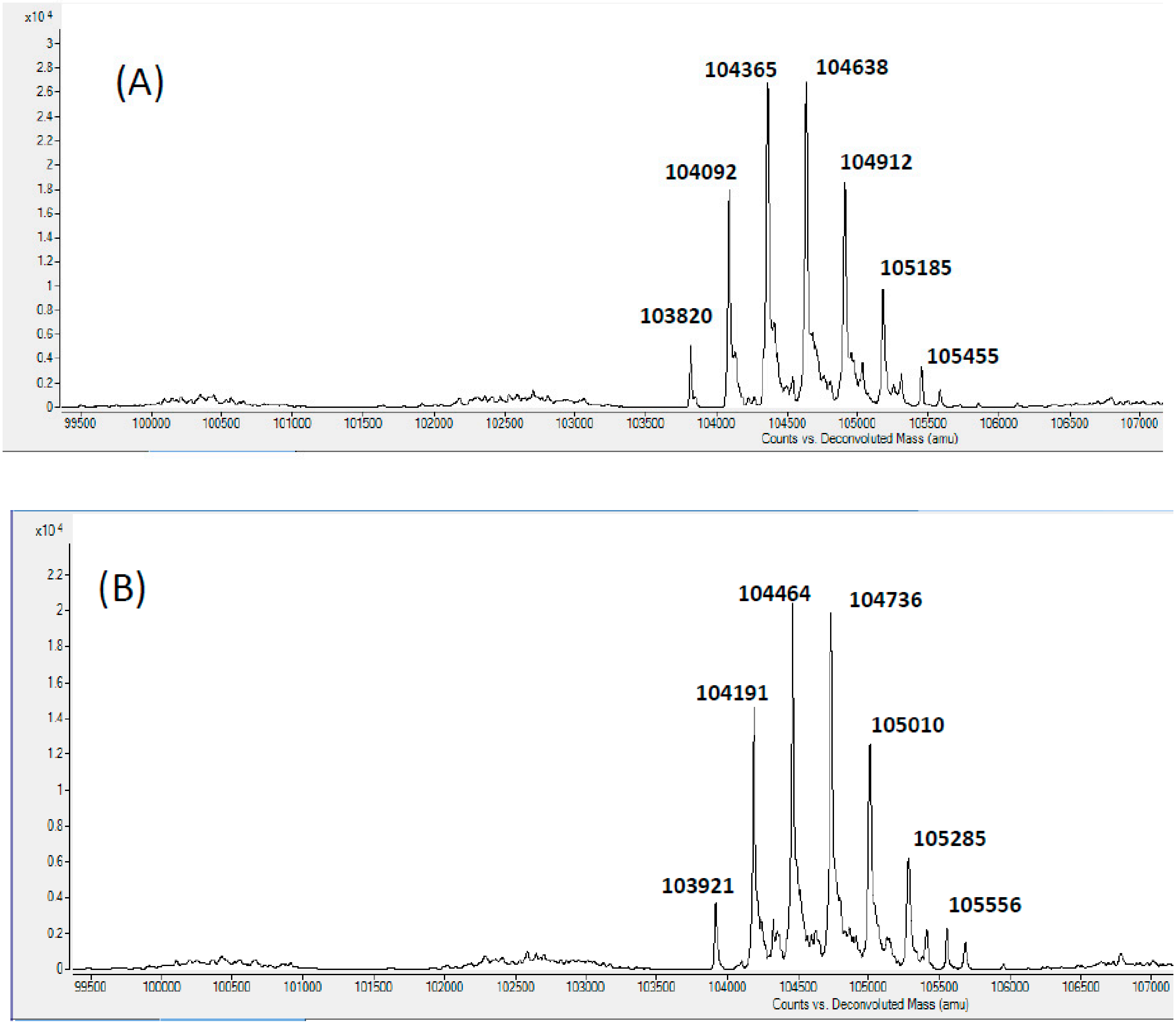


**Figure S1.** Random conjugation of NHS-azide. pMHC-Col_II (**A**) and pMHC-HA (**B**) were conjugated to NHS-PEG4-azide and analyzed by intact mass LC-MS to determine the DOL.

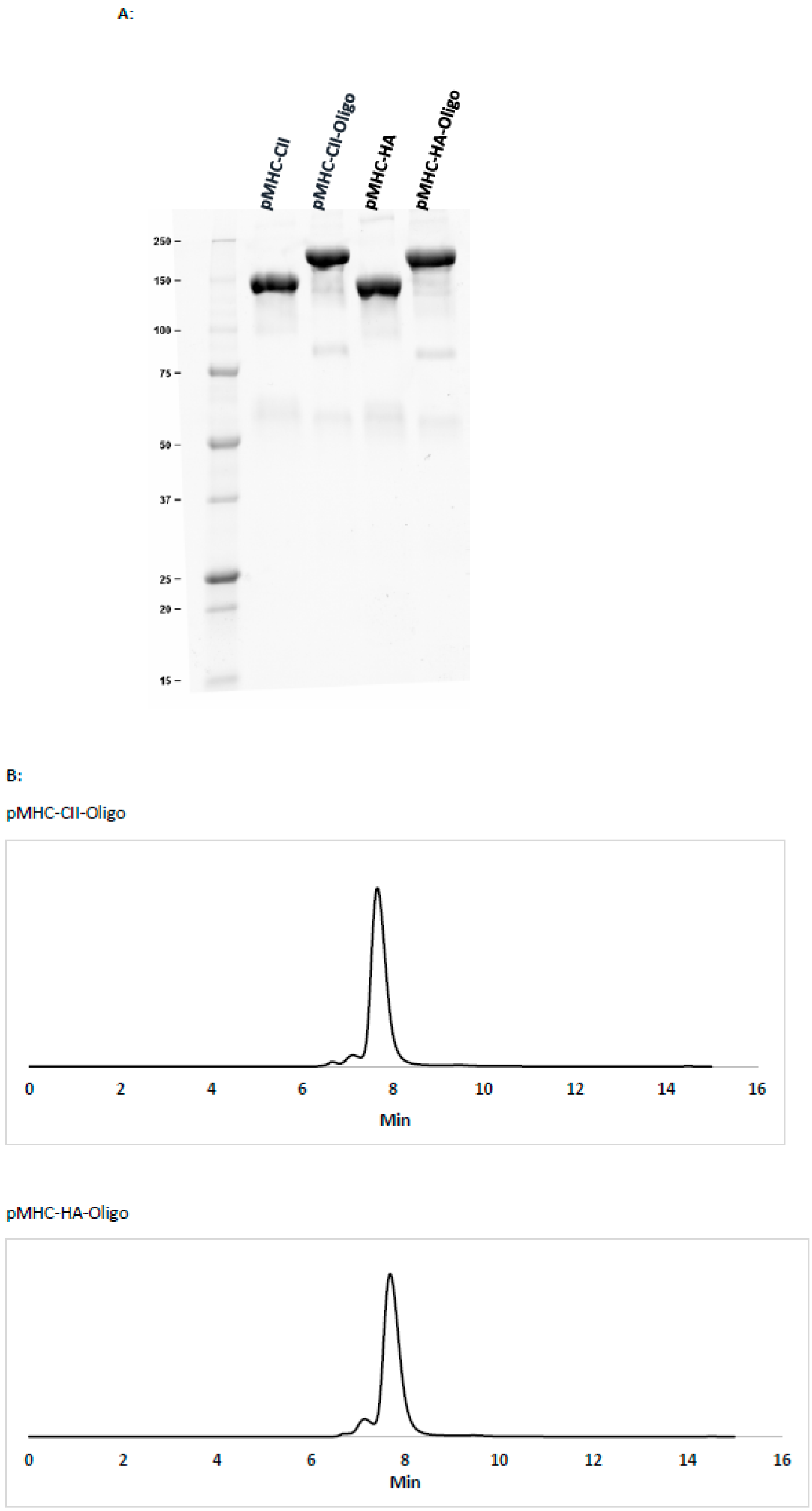


**Figure S2.** Characterization of pMHC-oligonucleotide conjugates. pMHC-oligonucleotide conjugates were characterized by SDS-PAGE (A) and analytical SEC (B).
